# Time trends in incidence and survival of small intestinal cancer in Sweden

**DOI:** 10.1093/bjsopen/zraa044

**Published:** 2021-01-08

**Authors:** K Landerholm

**Affiliations:** Department of Surgery, Ryhov County Hospital, Jönköping, Sweden; Department of Biomedical and Clinical Sciences, Linköping University, Linköping, Sweden

## Abstract

**Background:**

Small intestinal cancer is less common than some other gastrointestinal malignancies. Tumours of different histological types and anatomical sites of origin have therefore often been described together. The aim of this study was to investigate the epidemiology for each of the four main subtypes: duodenal adenocarcinoma (D-AC), duodenal neuroendocrine tumour (D-NET), jejunoileal adenocarcinoma (J/I-AC), and jejunoileal neuroendocrine tumour (J/I-NET).

**Methods:**

All patients with small intestinal cancer diagnosed between 1960 and 2015 were identified from the Swedish Cancer Register. The age-adjusted incidence rate with incidence rate ratios, as well as overall (OS) and net (NS) survival, were determined and temporal trends were analysed.

**Results:**

The incidence rate was highest for J/I-NET, with 9.98 clinical diagnoses per million in 2010–2015. Clinical diagnosis of D-AC increased more than 10-fold and surpassed J/I-AC as the second most common subtype. D-NET was by far the least common subtype. Diagnosis at autopsy became less common over time, whereas clinical diagnoses increased significantly for all four subtypes. All subtypes except J/I-AC affected men more often than women. The age distribution was similar between subtypes, although patients with adenocarcinomas were slightly older. Survival was generally much better for patients with NET than for those with adenocarcinoma. Both OS and NS showed a negative association with advancing age. Survival improved only for J/I-NET from a 5-year NS of 0.69 in the 1960s to 0.81 in 2010–2015.

**Conclusion:**

The incidence of small intestinal cancer is increasing, particularly for D-AC and in the elderly. Survival of patients with small intestinal cancer has improved only for J/I-NET over the last decades.

## Introduction

Small intestinal cancer represents less than 2.5 per cent of all gastrointestinal cancer^1^. This is probably one reason why the different types of small intestinal cancer have often been studied and discussed as one entity. Adenocarcinoma and neuroendocrine tumour (NET) constitute the two main epithelial cancer types; both may arise in either the duodenum or the jejunum or ileum. Most previous epidemiological studies grouped tumours of different histological types and/or anatomical sites of origin. This is inappropriate as adenocarcinoma and NET have distinctly different tumour biology, treatment and prognosis. Anatomical aspects also mean that duodenal tumours entail completely different surgical approaches compared with jejunoileal tumours.

National registries with complete coverage over many decades make it possible to investigate incidence and survival with temporal trends. The aim of the present study was to describe the epidemiology for each of the four main subtypes of small intestinal cancer: duodenal adenocarcinoma (D-AC), duodenal neuroendocrine tumour (D-NET), jejunoileal adenocarcinoma (J/I-AC) and jejunoileal neuroendocrine tumour (J/I-NET).

## Methods

This study was conducted and reported in accordance with the STROBE guidelines^2^ for cohort studies. The study was approved by the Regional Ethical Review Board at Linköping University (Dnr 2016-373-31).

### Data sources

A unique personal identity number is assigned to all permanent residents in Sweden. This identity number enables cross-linking between all registries held by authorities and healthcare providers.

The National Board of Health and Welfare’s Cancer Register has had full national coverage of all malignant tumours in Sweden since its initiation in 1958. Reporting to the registry is mandatory for all physicians and pathologists, and the registry contains data on how the tumour was diagnosed, its location, histopathological subtype and more. The registry has a completeness rate of 97.3 per cent^3^, and 98 per cent of all tumours and 100 per cent of small intestinal tumours are verified morphologically^4^.

The Cause of Death Register is also held by the National Board of Health and Welfare, and contains data including date of death.

Data on the composition and life expectancy of the Swedish population for each calendar year were retrieved from the Swedish Population Register provided by the government agency Statistics Sweden. These data were used for age standardization and calculations of net survival (NS).

### Study cohort

All patients diagnosed with a primary malignant small intestinal tumour between 1 January 1960 and 31 December 2015 were identified by ICD-7 codes 152.0–152.9 in the Cancer Register. Date of death for these individuals was obtained through linkage with the Cause of Death Register. Patients with histopathology codes 86 (carcinoid/NET) and 96 (adenocarcinoma) were eligible for the study. Additional refined morphology coding was introduced in 1993 (Systematized Nomenclature of Medicine (SNOMED) ICD-O/2, replaced in 2005 by SNOMED ICD-O/3), and a small number of mixed carcinoid/adenocarcinoma tumours were excluded from 1993 onwards. A minority of patients had more than one entry in the registry, and only the first entry was kept in the study cohort. Most cases of duplicate registration were on the same date or shortly afterwards.

### Definitions

In this study, the term small intestine refers to duodenum, jejunum and ileum. All analyses were performed for either the duodenum or the jejunum and ileum. Ampullary tumours were excluded from the study by their separate diagnosis code.

### Statistical analyses

The age- and sex-standardized incidence rates (IRs) of duodenal and jejunoileal NET and adenocarcinoma were calculated for 5-year strata of the study period using the population of Sweden in 2000 as reference. IRs are shown per million person-years, and incidence rate ratios (IRRs) are reported. Statistical significance between binary categorical variables, as well as linear trends in continuous and ordinal variables, were assessed by Poisson regression. Temporal trends for incidence were also assessed separately for each age group by means of Poisson regression.

TNM staging was introduced in the Cancer Register in 2005, and is reported in the study for adenocarcinoma from that year. A TNM staging system was proposed by the European Neuroendocrine Tumor Society for D-NETs in 2006^5^ and for J/I-NETs in 2007^6^, thereafter gradually adopted, and eventually implemented in the seventh edition of the UICC TNM classification of malignant tumours in 2009^7^. TNM staging for NETs is therefore reported from 2010 in this study. Time trends for the proportion of patients with complete TNM staging were calculated using the χ^2^ test for trend. The stage distribution between age groups was determined by Kendall’s τ rank correlation.

Overall survival (OS) was calculated using the Kaplan–Meier method, and univariable significance testing was performed by Cox proportional hazards regression. NS according to Pohar–Perme was estimated in strata using annual national data on expected survival for each sex and age at 1-year intervals^8^. Patients diagnosed at autopsy were excluded from all survival analyses. Risk factors for NS, including sex, age, time period and TNM stage, were assessed as excess mortality (EM) using univariable and multivariable Poisson regression.

All statistical analyses were carried out using STATA^®^/IC release 15.1 for Mac (StataCorp, College Station, TX, USA). All tests were two-tailed and *P* < 0.050 was considered statistically significant.

## Results

A total of 8751 patients with small intestinal NET or adenocarcinoma diagnosed between 1960 and 2015 were identified from the national registry. There were 6875 (78.6 per cent) clinical diagnoses and 1876 (21.4 per cent) diagnosed at autopsy.

### Incidence

#### Duodenal neuroendocrine tumour

**Fig. 1:**
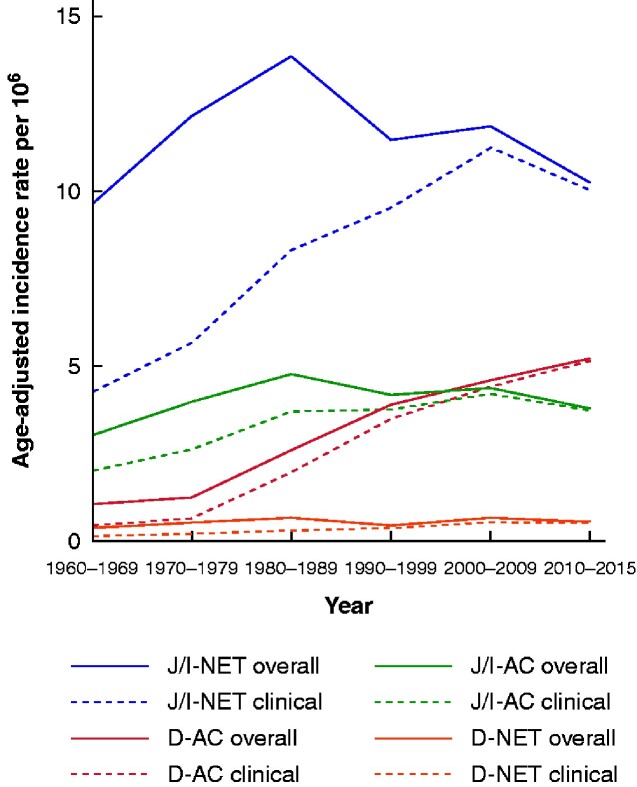
Temporal trends of age-adjusted clinical and overall (including autopsy) incidence of small intestinal cancer subtypes, 1960–2015 J/I-NET, jejunoileal neuroendocrine tumour; D-AC, duodenal adenocarcinoma; J/I-AC, jejunoileal adenocarcinoma; D-NET, duodenal neuroendocrine tumour.

**Fig. 2:**
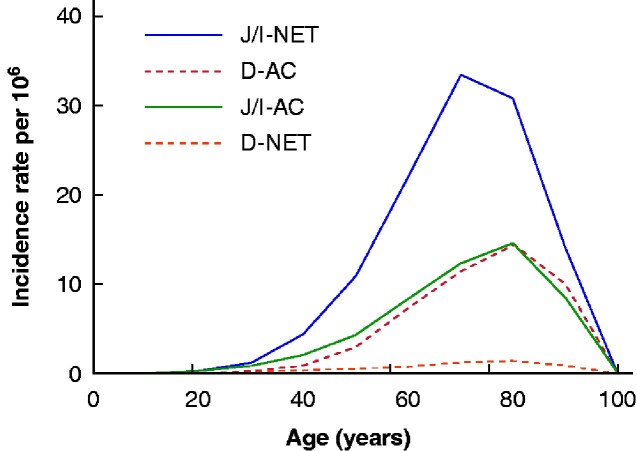
Incidence of small intestinal cancer subtypes at different ages J/I-NET, jejunoileal neuroendocrine tumour; D-AC, duodenal adenocarcinoma; J/I-AC, jejunoileal adenocarcinoma; D-NET, duodenal neuroendocrine tumour.

By far least common among the four subtypes, the age- and sex-standardized IR of D-NET was 0.34 (95 per cent c.i. 0.26 to 0.41) per 10^6^ population during the entire study period (*Table 1* and *Fig. 1*). D-NETs were more common in men than in women, and virtually non-existent before the age of 40 years, although the incidence increased with age (*Fig. 2*). The IR of clinically diagnosed D-NET increased significantly during the study period, whereas the IR remained unchanged when tumours detected at autopsy were included.

**Table 1:** Age-adjusted incidence of duodenal neuroendocrine tumour per million person-years

	Clinical diagnosis (*n *=* *161)			Clinical diagnosis + autopsy (*n *=* *237)		
	IR	IRR	***P*** ^†^	IR	IRR	***P*** ^†^
**Overall**	0.34 (0.26, 0.41)			0.50 (0.39-0.60)		
**Sex**			0.057			0.024
F	0.29 (0.19, 0.38)	Reference		0.42 (0.30, 0.55)	Reference	
M	0.39 (0.27, 0.51)	1.4 (1.0, 1.8)		0.57 (0.41, 0.73)	1.3 (1.0, 1.7)	
**Age (years)** ^*^			<0.001			<0.001
0–19	0	.		0		
20–39	0.09 (0.03, 0.15)	Reference		0.09 (0.03, 0.15)	Reference	
40–59	0.40 (0.29, 0.51)	4.3 (2.3, 8.2)		0.46 (0.34, 0.58)	5.0 (2.7, 9.3)	
60–79	0.89 (0.63, 1.14)	9.6 (5.2, 17.6)		1.31 (1.00, 1.62)	14.2 (7.8, 25.7)	
≥80	1.22 (0.68, 1.76)	13.2 (6.6, 26.6)		2.93 (2.10, 3.76)	31.6 (16.9, 59.0)	
**Period**			<0.001			0.135
1960–1969	0.13 (0.05, 0.22)	Reference		0.41 (0.23, 0.58)	Reference	
1970–1979	0.23 (0.10, 0.35)	1.7 (0.8, 3.6)		0.51 (0.32, 0.70)	1.2 (0.8, 2.0)	
1980–1989	0.29 (0.17, 0.41)	2.2 (1.0, 4.5)		0.66 (0.46, 0.83)	1.6 (1.0, 2.5)	
1990–1999	0.40 (0.26, 0.53)	3.0 (1.5, 6.0)		0.47 (0.32, 0.61)	1.1 (0.7, 1.8)	
2000–2009	0.54 (0.39, 0.69)	4.0 (2.1, 7.9)		0.65 (0.48, 0.80)	1.6 (1.0, 2.4)	
2010–2015	0.52 (0.34, 0.71)	3.9 (1.9, 8.0)		0.57 (0.37, 0.76)	1.4 (0.9, 2.3)	

Values in parentheses are 95 per cent confidence intervals.

*The incidence per age group is not age-adjusted. IR, incidence rate; IRR, incidence rate ratio.

^†^Poisson regression.

#### Duodenal adenocarcinoma

The IR of clinically diagnosed non-ampullary D-AC increased greatly from 0.47 (95 per cent c.i. 0.31 to 0.63) per 10^6^ population in the 1960s to 5.14 (4.57 to 5.72) per 10^6^ after 2010 (*Table 2* and *Fig. 1*). A diagnosis of D-AC at autopsy was relatively uncommon, particularly in recent years. These tumours appeared as often in men as in women, but IR was strongly associated with advancing age (*Fig. 2*).

**Table 2:** Age-adjusted incidence of duodenal adenocarcinoma per million person-years

	Clinical diagnosis(*n *=* *1281)			Clinical diagnosis + autopsy(*n *=* *1462)		
	IR	IRR	***P*** ^†^	IR	IRR	***P*** ^†^
**Overall**	2.68 (2.06, 3.29)			3.05 (2.39, 3.72)		
**Sex**			0.006			0.003
F	2.47 (1.94, 3.82)	Reference		2.82 (1.96, 3.68)	Reference	
M	2.88 (1.67, 3.27)	1.2 (1.0, 1.3)		3.29 (2.26, 4.31)	1.2 (1.1, 1.3)	
**Age (years)** ^*^			<0.001			<0.001
0–19	0.02 (0.01, 0.04)	0.1 (0.0, 0.4)		0.02 (0.01, 0.04)	0.1 (0.0, 0.4)	
20–39	0.16 (0.06, 0.26)	Reference		0.17 (0.07, 0.27)	Reference	
40–59	1.80 (1.28, 2.32)	11.1 (7.1, 17.4)		1.95 (1.42, 2.49)	11.5 (7.4, 17.8)	
60–79	8.99 (7.05, 10.9)	55.5 (36.0, 85.6)		10.2 (8.24, 12.1)	60.0 (39.3, 91.5)	
≥80	13.7 (9.81, 17.6)	84.7 (54.3, 132.1)		16.8 (13.1, 20.4)	98.7 (64.1, 152.1)	
**Period**			<0.001			<0.001
1960–1969	0.47 (0.31, 0.63)	Reference		1.05 (0.78, 1.33)	Reference	
1970–1979	0.67 (0.48, 0.85)	1.4 (0.9, 2.1)		1.25 (0.98, 1.51)	1.2 (0.9, 1.6)	
1980–1989	1.97 (1.66, 2.27)	4.2 (2.9, 6.0)		2.59 (2.30, 3.03)	2.5 (2.0, 3.3)	
1990–1999	3.50 (3.10, 3.89)	7.4 (5.2, 10.4)		3.91 (3.49, 4.32)	3.7 (2.9, 4.7)	
2000–2009	4.40 (3.97, 4.83)	9.3 (6.6, 13.1)		4.58 (4.14, 5.02)	4.3 (3.4, 5.5)	
2010–2015	5.14 (4.57, 5.72)	10.9 (7.7, 15.3)		5.24 (4.66, 5.82)	5.0 (3.9, 6.3)	

Values in parentheses are 95 per cent confidence intervals.

*The incidence per age group is not age-adjusted. IR, incidence rate; IRR, incidence rate ratio.

^†^Poisson regression.

#### Jejunoileal neuroendocrine tumour

The most common subtype of malignant small intestinal cancer was J/I-NET, with an IR of 8.07 (95 per cent c.i. 6.57 to 9.57) per 10^6^ population for clinically detected cases (*Table 3*). Men were affected more often (IRR 1.2, 95 per cent c.i. 1.1 to 1.3), as were older people, with a notably increased IRR above 60 years of age (*Fig. 2*). The IR for clinical cases more than doubled between 1960 and 2015, whereas the IR including autopsy cases did not change at all over this interval (*Fig. 1*).

**Table 3:** Age-adjusted incidence of jejunoileal neuroendocrine tumour per million person-years

	Clinical diagnosis(*n *=* *3866)			Clinical diagnosis + autopsy(*n *=* *5223)		
	IR	IRR	***P*** ^†^	IR	IRR	***P*** ^†^
**Overall**	8.07 (6.57, 9.57)			10.90 (8.91, 12.90)		
**Sex**			<0.001			<0.001
F	7.35 (5.45, 9.26)	Reference		9.58 (7.18, 12.00)	Reference	
M	8.79 (6.46, 11.11)	1.2 (1.1, 1.3)		12.20 (9.04, 15.40)	1.3 (1.2, 1.3)	
**Age (years)** ^*^			<0.001			<0.001
0–19	0.02 (0, 0.05)	0.03 (0.01, 0.11)		0.02 (0, 0.05)	0.03 (0.01, 0.10)	
20–39	0.72 (0.47, 0.97)	Reference		0.76 (0.50, 1.02)	Reference	
40–59	7.51 (6.15, 8.87)	10.5 (8.4, 12.9)		8.31 (6.82, 9.80)	10.9 (8.8, 13.4)	
60–79	26.7 (23.3, 30.1)	37.2 (30.2, 45.7)		35.8 (31.8, 39.9)	46.9 (38.4, 57.3)	
≥80	28.5 (22.2, 34.8)	39.7 (31.9, 49.5)		52.9 (45.0, 60.8)	69.2 (56.3, 85.1)	
**Period**			<0.001			0.647
1960–1969	4.25 (3.74, 4.75)	Reference		9.64 (8.78, 10.50)	Reference	
1970–1979	5.68 (5.13, 6.23)	1.3 (1.2, 1.5)		12.16 (11.29, 13.04)	1.3 (1.1, 1.4)	
1980–1989	8.35 (7.71, 8.98)	2.0 (1.7, 2.2)		13.89 (13.05, 14.74)	1.4 (1.3-1.6)	
1990–1999	9.53 (8.88, 10.19)	2.2 (2.0, 2.6)		11.46 (10.74, 12.18)	1.2 (1.1, 1.3)	
2000–2009	11.07 (10.39, 11.75)	2.6 (2.3, 3.0)		11.87 (11.12, 12.58)	1.2 (1.1, 1.4)	
2010–2015	9.98 (9.18, 10.78)	2.4 (2.1, 2.7)		10.28 (9.47, 11.09)	1.1 (1.0, 1.2)	

Values in parentheses are 95 per cent confidence intervals.

*The incidence per age group is not age-adjusted. IR, incidence rate; IRR, incidence rate ratio.

^†^Poisson regression.

#### Jejunoileal adenocarcinoma

Most J/I-ACs were detected clinically, and the IR did not differ between men and women (*Table 4*). The incidence nearly doubled during the study period and was associated with age (*Figs 1* and 2).

**Table 4:** Age-adjusted incidence of jejunoileal adenocarcinoma per million person-years

	Clinical diagnosis(*n *=* *1567)			Clinical diagnosis + autopsy(*n *=* *1829)		
	IR	IRR	***P*** ^†^	IR	IRR	***P*** ^†^
**Overall**	3.27 (2.70, 3.84)			3.82 (3.16, 4.47)		
**Sex**			0.648			0.455
F	3.23 (2.43, 4.03)	Reference		3.75 (2.84, 4.66)	Reference	
M	3.31 (2.50, 4.11)	1.0 (0.9, 1.1)		3.88 (2.94, 4.83)	1.0 (0.9, 1.1)	
**Age (years)** ^*^			<0.001			<0.001
0–19	0	.		0	.	
20–39	0.53 (0.35, 0.72)	Reference		0.57 (0.37, 0.77)	Reference	
40–59	3.10 (2.60, 3.59)	5.8 (4.5, 7.5)		3.40 (2.88, 3.92)	6.0 (4.6, 7.6)	
60–79	9.95 (8.78, 11.1)	18.7 (14.6, 23.9)		11.5 (10.2, 12.8)	20.2 (15.9, 25.6)	
≥80	13.6 (11.6, 15.7)	25.6 (19.6, 33.4)		18.0 (15.8, 20.2)	31.5 (24.5, 40.5)	
**Period**			<0.001			0.010
1960–1969	2.03 (1.66, 2.41)	Reference		3.02 (2.54, 3.51)	Reference	
1970–1979	2.63 (2.24, 3.02)	1.3 (1.1, 1.6)		3.99 (3.49, 4.49)	1.3 (1.1, 1.6)	
1980–1989	3.70 (3.27, 4.13)	1.8 (1.5, 2.2)		4.76 (4.27, 5.26)	1.6 (1.3, 1.9)	
1990–1999	3.76 (3.35, 4.17)	1.9 (1.5, 2.2)		4.16 (3.73, 4.59)	1.4 (1.2, 1.6)	
2000–2009	4.24 (3.82, 4.66)	2.1 (1.7, 2.5)		4.39 (3.97, 4.82)	1.5 (1.2, 1.7)	
2010–2015	3.74 (3.24, 4.23)	1.8 (1.5, 2.3)		3.77 (3.27, 4.27)	1.2 (1.0, 1.5)	

Values in parentheses are 95 per cent confidence intervals.

*The incidence per age group is not age-adjusted. IR, incidence rate; IRR, incidence rate ratio.

^†^Poisson regression.

#### Temporal incidence trend and age

The increased incidence of clinically diagnosed small intestinal cancer occurred particularly within the older population. The IR of each of the four subtypes increased significantly more in the elderly than in younger patients (data not shown).

#### TNM stage

TNM staging after its introduction in the registries is detailed in *Table 5*. A large proportion of tumours lacked data on TNM, especially among patients with NET. The proportion of patients with NET who had complete TNM staging registered increased significantly when a separate TNM classification for NETs was introduced in 2010, but did not improve thereafter. The proportion of patients with adenocarcinoma who had complete TNM staging did not change after it was introduced in the register in 2005. At least 19.6 per cent of patients with D-AC had stage 3 disease, and 24.2 per cent had stage 4. Corresponding rates for patients with J/I-AC were 17.9 and 19.3 per cent respectively. The proportion of patients with complete TNM staging in the register did not differ between age groups, and neither was the stage distribution for those with a complete TNM stage associated with age.

**Table 5:** Reported TNM stage from 2010 for neuroendocrine tumours and from 2005 for adenocarcinomas

TNM stage	NET		Adenocarcinoma	
	Duodenum (*n *=* *31)	Jejunum/ileum (*n* = 617)	Duodenum (*n* = 565)	Jejunum/ileum (*n* = 431)
1	5 (16)	30 (4.9)	51 (9.0)	24 (5.6)
2	0 (0)	36 (5.8)	75 (13.3)	133 (30.9)
3	1 (3)	199 (32.3)	111 (19.6)	77 (17.9)
4	2 (6)	144 (23.3)	137 (24.2)	83 (19.3)
Unknown	23 (74)	208 (33.7)	191 (33.8)	114 (26.5)

Values in parentheses are percentages. NET, neuroendocrine tumour.

### Survival

#### Duodenal neuroendocrine tumour

**Fig. 3:**
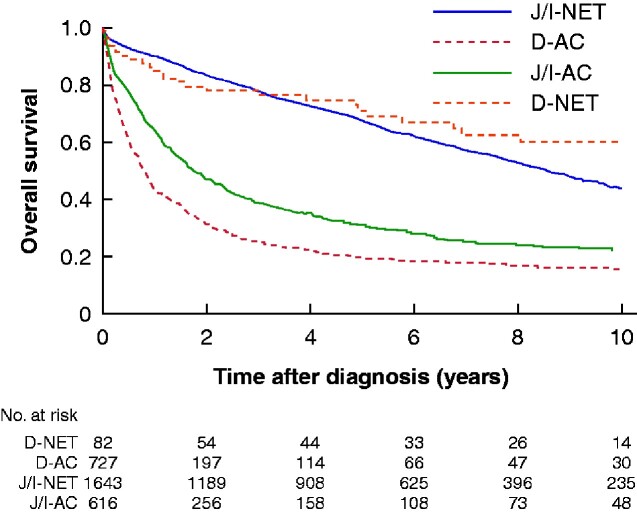
Kaplan–Meier curves for overall survival in small intestinal cancer subtypes of patients diagnosed from 2000 onwards later J/I-NET, jejunoileal neuroendocrine tumour; D-AC, duodenal adenocarcinoma; J/I-AC, jejunoileal adenocarcinoma; D-NET, duodenal neuroendocrine tumour.

**Fig. 4:**
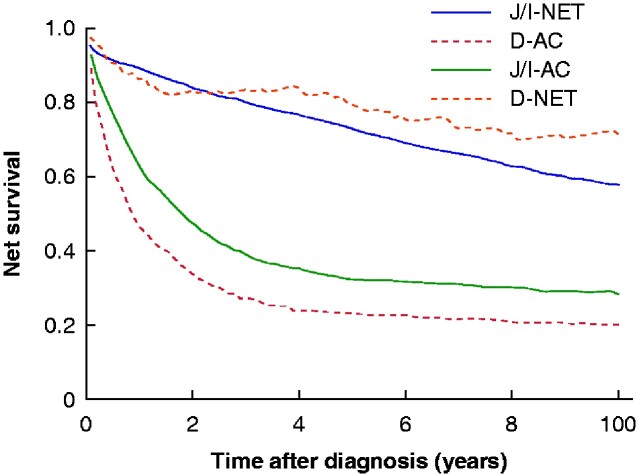
Net survival in small intestinal cancer subtypes J/I-NET, jejunoileal neuroendocrine tumour; D-AC, duodenal adenocarcinoma; J/I-AC, jejunoileal adenocarcinoma; D-NET, duodenal neuroendocrine tumour.

The 5-year OS for D-NET was 0.68 (95 per cent c.i. 0.60 to 0.75) and 5-year NS was 0.79 (0.68 to 0.87) (*Table 6* and *Figs 3* and 4). Survival did not differ between men and women, and NS was not associated with age. Neither OS nor NS changed during the study period.

**Table 6:** Survival in patients with duodenal neuroendocrine tumour, with risk factors

	5-year overall survival	***P*** ^*^	5-year net survival	***P*** ^*^
**Overall**	0.68 (0.60, 0.75)		0.79 0.68, 0.87	
**Sex**		0.162		0.110
F	0.59 (0.46, 0.70)		0.71 (0.52, 0.84)	
M	0.75 (0.64, 0.83)		0.85 (0.69, 0.93)	
**Age (years)**		<0.001		0.139
0–39	0.91 (0.52, 0.99)		0.92 (0.50, 0.99)	
40–59	0.75 (0.60, 0.85)		0.76 (0.61, 0.87)	
60–79	0.68 (0.56, 0.78)		0.81 (0.63, 0.90)	
≥80	0.39 (0.18, 0.60)		0.73 (0.12, 0.95)	
**Period**		0.436		0.845
1960–1969	0.80 (0.41, 0.95)		0.86 (0.27, 0.98)	
1970–1979	0.62 (0.31, 0.82)		0.89 (0.00, 1.00)	
1980–1989	0.61 (0.38, 0.77)		0.69 (0.41, 0.86)	
1990–1999	0.68 (0.49, 0.81)		0.79 (0.50, 0.92)	
2000–2009	0.69 (0.54, 0.79)		0.76 (0.57, 0.87)	
2010–2015	0.77 (0.49, 0.90)		0.90 (0.01, 1.00)	
**TNM stage**		–		–
1–2	n.a.		n.a.	
3	n.a.		n.a.	
4	n.a.		n.a.	
Unknown	n.a.		n.a.	

Values in parentheses are 95 per cent confidence intervals.

*Cox proportional hazards regression.

#### Duodenal adenocarcinoma

Survival in non-ampullary D-AC was poor, with 5-year OS of 0.21 (95 per cent c.i. 0.18 to 0.23) and NS of 0.23 (0.20 to 0.26) (*Table 7* and *Figs 3* and *4*). Survival did not differ between men and women, with no improvement since the 1960s. Advancing age was associated not only with OS but also with much worse NS.

**Table 7:** Survival in patients with duodenal adenocarcinoma, with risk factors

	5-year overall survival	***P*** ^*^	5-year net survival	***P*** ^*^
**Overall**	0.21 (0.18, 0.23)		0.23 (0.20, 0.26)	
**Sex**		0.683		0.958
F	0.20 (0.17, 0.24)		0.23 (0.19, 0.27)	
M	0.23 (0.18, 0.24)		0.23 (0.19, 0.26)	
**Age (years)**		<0.001		<0.001
0–39	0.39 (0.20, 0.58)		0.39 (0.20, 0.58)	
40–59	0.36 (0.29, 0.42)		0.37 (0.30, 0.43)	
60–79	0.22 (0.19, 0.25)		0.24 (0.21, 0.28)	
≥80	0.02 (0.01, 0.05)		0.04 (0.01, 0.08)	
**Period**		0.491		0.469
1960–1969	0.26 (0.13, 0.42)		0.29 (0.14, 0.45)	
1970–1979	0.23 (0.13, 0.35)		0.24 (0.13, 0.38)	
1980–1989	0.23 (0.17, 0.29)		0.26 (0.19, 0.34)	
1990–1999	0.19 (0.15, 0.23)		0.21 (0.17, 0.26)	
2000–2009	0.18 (0.15, 0.22)		0.20 (0.16, 0.24)	
2010–2015	0.25 (0.19, 0.31)		0.27 (0.21, 0.34)	
**TNM stage**		<0.001		<0.001
1–2	0.44 (0.35, 0.53)		0.50 (0.39, 0.60)	
3	0.32 (0.23, 0.43)		0.34 (0.24, 0.45)	
4	0.03 (0.00, 0.10)		0.03 (0.00, 0.11)	
Unknown	0.12 (0.07, 0.18)		0.13 (0.08, 0.19)	

Values in parentheses are 95 per cent confidence intervals.

*Cox proportional hazards regression.

#### Jejunoileal neuroendocrine tumour

The 5-year OS for J/I-NET was 0.62 (95 per cent c.i. 0.61 to 0.64) and 5-year NS was 0.73 (0.71 to 0.75) (*Table 8* and *Figs 3* and *4*). No difference was observed between men and women. NS was worse in older patients. Both OS and NS improved significantly during the study period: 5-year NS increased from 0.69 (0.62 to 0.75) in the 1960s to 0.81 (0.74 to 0.86) after 2010.

**Table 8:** Survival in patients with jejunoileal neuroendocrine tumour, with risk factors

	5-year overall survival	***P*** ^*^	5-year net survival	***P*** ^*^
**Overall**	0.62 (0.61, 0.64)		0.73 (0.71, 0.75)	
**Sex**		0.842		0.217
F	0.63 (0.61, 0.65)		0.72 (0.69, 0.75)	
M	0.62 (0.60, 0.64)		0.74 (0.71, 0.76)	
**Age (years)**		<0.001		<0.001
0–39	0.93 (0.85, 0.96)		0.93 (0.85, 0.97)	
40–59	0.80 (0.78, 0.83)		0.83 (0.80, 0.85)	
60–79	0.60 (0.58, 0.62)		0.70 (0.67, 0.72)	
≥80	0.35 (0.31, 0.40)		0.65 (0.56, 0.73)	
**Period**		0.032		<0.001
1960–1969	0.61 (0.55, 0.66)		0.69 (0.62, 0.75)	
1970–1979	0.56 (0.51, 0.61)		0.66 (0.60, 0.71)	
1980–1989	0.59 (0.55, 0.62)		0.69 (0.65, 0.74)	
1990–1999	0.60 (0.57, 0.63)		0.70 (0.65, 0.74)	
2000–2009	0.68 (0.63, 0.69)		0.79 (0.75, 0.82)	
2010–2015	0.71 (0.66, 0.76)		0.81 (0.74, 0.86)	
**TNM stage**		0.026		0.077
1–2	0.78 (0.63, 0.88)		0.91 (0.59, 0.98)	
3	0.81 (0.73, 0.87)		0.92 (0.77, 0.98)	
4	0.57 (0.45, 0.67)		0.65 (0.50, 0.76)	
Unknown	0.71 (0.62, 0.78)		0.80 (0.68, 0.88)	

Values in parentheses are 95 per cent confidence intervals.

*Cox proportional hazards regression.

#### Jejunoileal adenocarcinoma

The 5-year NS in J/I-AC was 0.32 (95 per cent c.i. 0.30 to 0.35), and was worse for men compared with women (*Table 9* and *Figs 3* and *4*). The 5-year NS was only 0.39 (0.27 to 0.50) among the youngest patients, yet worse for older patients. Survival had not changed since 1960.

**Table 9:** Survival in patients with jejunoileal adenocarcinoma, with risk factors

	5-year overall survival	***P*** ^*^	5-year net survival	***P*** ^*^
**Overall**	0.28 (0.26, 0.30)		0.32 (0.30, 0.35)	
**Sex**		0.005		0.027
F	0.30 (0.27, 0.33)		0.34 (0.30, 0.38)	
M	0.26 (0.23, 0.29)		0.31 (0.27, 0.34)	
**Age (years)**		<0.001		<0.001
0–39	0.39 (0.27, 0.50)		0.39 (0.27, 0.50)	
40–59	0.35 (0.30, 0.40)		0.36 (0.31, 0.41)	
60–79	0.28 (0.25, 0.31)		0.33 (0.29, 0.36)	
≥80	0.13 (0.09, 0.17)		0.24 (0.16, 0.33)	
**Period**		0.429		0.332
1960–1969	0.33 (0.25, 0.41)		0.40 (0.30, 0.49)	
1970–1979	0.25 (0.19, 0.31)		0.31 (0.23, 0.38)	
1980–1989	0.25 (0.21, 0.31)		0.29 (0.23, 0.35)	
1990–1999	0.25 (0.21, 0.30)		0.30 (0.24, 0.36)	
2000–2009	0.31 (0.26, 0.35)		0.35 (0.29, 0.40)	
2010–2015	0.29 (0.21, 0.38)		0.33 (0.23, 0.43)	
**TNM stage**		<0.001		<0.001
1–2	0.48 (0.39, 0.56)		0.56 (0.45, 0.66)	
3	0.38 (0.26, 0.50)		0.43 (0.29, 0.56)	
4	0.06 (0.02, 0.15)		0.07 (0.02, 0.15)	
Unknown	0.25 (0.17, 0.35)		0.28 (0.18, 0.38)	

Values in parentheses are 95 per cent confidence intervals.

*Cox proportional hazards regression.

The incidence of small intestinal cancer is increasing, especially for duodenal adenocarcinoma; jejunoileal neuroendocrine tumour remains the most common subtype. Both overall and net survival have improved only for patients with jejunal or ileal neuroendocrine tumours.

#### TNM stage and survival

TNM stage was not available for a sufficient number of patients with D-NET to allow any analysis. For the other three subtypes of small intestinal cancer, TNM stage was clearly associated with both OS and NS (*Tables 7–9*). As expected, the group with unknown TNM stage appeared as a mixture of the stages.

#### Multivariable analysis of risk factors for survival

All risk factors for NS identified from univariable analysis remained the same after adjustment. The improvement in NS for J/I-NET was even more pronounced after adjustment, with EM of 0.49 (0.35 to 0.70) in the last decade compared with that in the 1960s. Age at diagnosis affected NS much more for NETs compared with adenocarcinomas; EM was 11.08 (5.30 to 23.16) in the oldest group of patients with J/I-NET, but only 1.67 (1.19 to 2.35) in those with J/I-AC. Multivariable analysis was not possible for D-NET owing to the small number of patients.

## Discussion

This large population-based cohort study of the epidemiology of small intestinal cancer was made possible by high-quality national registries. A number of publications^9–13^ have described the epidemiology of small intestinal cancer in the USA, based on data from the Surveillance, Epidemiology, and End Results (SEER) database, but the present study represents the largest cohort from any other country. Many previous studies have been less detailed regarding the histopathological diagnosis, and tumours originating in the duodenum, jejunum and ileum have often been treated as one entity. At times it has not been clear whether duodenal tumours were included or not. In the present study, the four subtypes of small intestinal cancer (NET and adenocarcinoma of duodenum and jejunum/ileum) have been treated separately as they constitute distinctly different diseases for both histopathological and anatomical purposes, with implications for their prognosis and treatment— not least surgery. These differences are clear from the results of this study.

It is also often unclear from previous studies whether tumours diagnosed at autopsy were included; most often they probably were not. In this study, the incidence of clinically diagnosed small intestinal cancer as well as the overall incidence including autopsy findings were included, enabling a deeper understanding of temporal trends. In general, post mortem diagnoses were more common early in the study period and gradually declined between 1960 and 2015. This was partly due to a greatly decreased frequency of autopsies among the deceased in Sweden during the long study period. The increase in clinical diagnoses of small intestinal cancer relative to autopsies is probably also explained by improved diagnostic capability. Symptomatic patients are thereby more likely to receive a correct diagnosis. In addition, asymptomatic patients are now more likely to have diagnosis as an incidental finding.

This hypothesis is particularly valid for NETs, which are often described as potentially indolent tumours. Studies from Malmö County during 1958–1982, with an autopsy frequency reaching 87 per cent of all deceased persons found an extremely high incidence of 53 small intestinal NETs per million person-years^14^^,^^15^. The vast majority of these patients had been asymptomatic. Although the incidence was not quite as high in the present study, a majority of NETs were diagnosed at autopsy in the 1960s, also at a national level, yet only a small fraction in recent years. The incidence of clinically diagnosed J/I-NET more than doubled during the study period, and that of D-NETs nearly quadrupled. At the same time, the overall incidence including autopsies remained unchanged for both NET subtypes. This makes it difficult to discern whether a true increase has occurred in the incidence of small intestinal NET. What would support a true increase is that most patients with a clinical diagnosis in fact had symptoms leading to their diagnosis, as shown in a previous study^16^.

In accordance with the present results, a steeply increasing incidence of J/I-NET over the last decades has been described from several countries^10^^,^^16–20^. As an example, the incidence of ileal NET doubled between 1973 and 2002 in the SEER database^10^, reaching 6.7 per million in 2000–2004^11^. The corresponding rate in the present study was 11.07 per million, and previous studies^16^^,20,^^21^ from Scandinavia have consistently shown a higher incidence than that in other areas.

Few population-based studies have focused specifically on D-NET. As an exception and in accordance with the present results, Fitzgerald and colleagues^9^ found a 4-fold increase in the incidence of D-NET in the SEER database between 1983 and 2010. The incidence of D-NET was reported at 1.9 per million in 2000–2004 from the SEER database^11^ and at 1.4 per million in a smaller study from Norway^21^. There is no obvious explanation for the lower incidence of just over 0.50 per million after 2000 in the Swedish Cancer Register, but the present results are in keeping with a previous report^20^ using data from the same registry.

Adencocarcinomas were diagnosed relatively less often at autopsy compared with NETs, and virtually no adenocarcinomas have been diagnosed after death in recent years. The different pattern compared with NETs probably reflects that patients with adenocarcinoma generally do not remain asymptomatic for any length of time, owing to the more aggressive nature of the disease. For this reason it seems likely that the increased incidence of adenocarcinoma represents a true change, considering that the mortality rate is high and symptomatic patients would have had an autopsy if the diagnosis had not been established already at the time of death.

The most remarkable temporal trend was the notable increase in D-AC, with an 11-fold increased incidence of clinical cases and a 5-fold increase in the overall incidence. There has been no change in coding that could explain this development, and a true increase for unknown reasons seems probable. Proton pump inhibitors have been found to contribute to the development of gastric cancer, but the suggested mechanisms for that do not easily translate to the duodenum^22^^,^^23^.

The rise in incidence of non-ampullary D-AC observed in the present study is in keeping with a similar development in other European countries, including Denmark^24^ and the Netherlands^25^. From the SEER database, an incidence of 3.7 per million was reported for 1992–2006^13^, closely resembling the present results. Duodenal adenocarcinoma has been more common than J/I-AC for a longer time in the USA^12^^,^^13^, but the increase in D-AC has not been quite as striking over time as in Sweden.

Small intestinal cancer of both subtypes has repeatedly been found to affect men more often than women^9^^,^^11^^,^^13^. In the present study, three of the four subtypes of small intestinal cancer were more common in men than in women; only the incidence of J/I-AC did not differ significantly. Although the IR differed greatly between the four subtypes, the age distribution of clinically diagnosed tumours was similar, with occasional cases before the age of 40 years followed by a peak around 70 years for NET and 80 years for adenocarcinoma. A similar distribution has been described from the SEER database^13^.

NS is superior to OS to describe temporal trends in survival, as it adjusts for demographic changes in the general population. It is equally remarkable and disappointing that neither OS nor NS improved for three of the four cancer subtypes, particularly as better imaging would presumably lead to incidental findings as well as earlier established diagnoses for symptomatic patients, thereby allowing longer follow-up by lead-time bias. More encouraging was the statistically significant increase in OS and NS between 1960 and 2015 for patients with J/I-NET. Lead-time bias may contribute, but hopefully the results mirror the extensive development of treatment options, including somatostatin analogues and peptide receptor radionuclide therapy.

Previous data on the development of survival over time for the four subtypes are sparse; instead, trends for the entire small intestine including the duodenum are frequently reported. As an exception, Zar and co-workers^26^^,^^27^ reported temporal trends for survival in duodenal and jejunoileal adenocarcinoma (1960–1988) and NET (1960–2000) in the Swedish Cancer Register. Improved survival was also reported only for J/I-NET in those studies^26,27^. Improved survival was also confirmed in the present author’s previous detailed regional study of J/I-NETs^28^. A limited number of studies^13^^,^^24^^,^^29–31^ from other countries separately reported the survival specified for one or more of the four subtypes, all in keeping with the present study. None described temporal trends.

Registry studies have limitations. First, there is the risk of missing or erroneous data, but, as already mentioned, the Swedish Cancer Register has been validated and found to be of high standard^3^. Second, some relevant data may not be available from the register; TNM stage, for instance, was not reported until 2005. Third, coding of disease may change over time. SNOMED ICD-O/2 was introduced in 1993 and allowed the exclusion of a small number of mixed carcinoid/adenocarcinoma tumours, but no major amendments were otherwise made during the long study interval.

## Funding

Futurum, Academy for Health and Care, Region Jönköping County

## References

[1] Swedish National Board of Health and Welfare. *Statistics on Cancer.* https://www.socialstyrelsen.se/statistik-och-data/statistik/statistikamnen/cancer/ (accessed 18 May 2020)

[2] von Elm E , Altman DG, Egger M, Pocock SJ, Gøtzsche PC, Vandenbroucke JP; STROBE Initiative. The Strengthening the Reporting of Observational Studies in Epidemiology (STROBE) statement: guidelines for reporting observational studies. Lancet 2007;370:1453–1457 18064739 10.1016/S0140-6736(07)61602-X

[3] Barlow L , Westergren K, Holmberg L, Talbäck M. The completeness of the Swedish Cancer Register: a sample survey for year 1998. Acta Oncol 2009;48:27–33 18767000 10.1080/02841860802247664

[4] Swedish National Board of Health and Welfare. Cancer incidence in Sweden in 2009. https://www.socialstyrelsen.se/globalassets/sharepoint-dokument/artikelkatalog/statistik/2010-12-17.pdf (accessed 18 May 2020)

[5] Rindi G , Klöppel G, Alhman H, Caplin M, Couvelard A, de Herder WW et al; all other Frascati Consensus Conference participants; European Neuroendocrine Tumor Society (ENETS). TNM staging of foregut (neuro)endocrine tumors: a consensus proposal including a grading system. Virchows Arch 2006;449:395−401 16967267 10.1007/s00428-006-0250-1 PMC1888719

[6] Rindi G , Klöppel G, Couvelard A, Komminoth P, Körner M, Lopes JM et al; all other Frascati Consensus Conference participants; European Neuroendocrine Tumor Society (ENETS). TNM staging of midgut and hindgut (neuro) endocrine tumors: a consensus proposal including a grading system. Virchows Arch 2007;451:757−762 17674042 10.1007/s00428-007-0452-1

[7] Sobin LH , Gospodarowicz MK, Wittekind C (eds). UICC International Union Against Cancer: TNM Classification of Malignant Tumours (7th edn). Chichester: Wiley–Blackwell, 2009

[8] Dickman PW , Coviello E. Estimating and modeling relative survival. Stata J 2015;15:186–215

[9] Fitzgerald TL , Dennis SO, Kachare SD, Vohra NA, Zervos EE. Increasing incidence of duodenal neuroendocrine tumors: incidental discovery of indolent disease? Surgery 2015;158:466–471 26013986 10.1016/j.surg.2015.03.042

[10] Modlin IM, , Champaneria MC, Chan AK, Kidd M. A three-decade analysis of 3911 small intestinal neuroendocrine tumors: the rapid pace of no progress. Am J Gastroenterol 2007;102:1464−1473 17391319 10.1111/j.1572-0241.2007.01185.x

[11] Yao JC , Hassan M, Phan A, Dagohoy C, Leary C, Mares JE et al One hundred years after ‘carcinoid’: epidemiology of and prognostic factors for neuroendocrine tumors in 35 825 cases in the United States. J Clin Oncol 2008;26:3063−3072 18565894 10.1200/JCO.2007.15.4377

[12] Bilimoria KY , Bentrem DJ, Wayne JD, Ko CY, Bennett CL, Talamonti MS. Small bowel cancer in the United States: changes in epidemiology, treatment, and survival over the last 20 years. Ann Surg 2009;249:63−71 19106677 10.1097/SLA.0b013e31818e4641

[13] Qubaiah O , Devesa SS, Platz CE, Huycke MM, Dores GM. Small intestinal cancer: a population-based study of incidence and survival patterns in the United States, 1992 to 2006. Cancer Epidemiol Biomarkers Prev 2010;19:1908–1918 20647399 10.1158/1055-9965.EPI-10-0328 PMC2919612

[14] Berge T , Linell F. Carcinoid tumours. Frequency in a defined population during a 12-year period. Acta Pathol Microbiol Scand 1976;84:322−330 961424

[15] Eriksson J , Norlén O, Ögren M, Garmo H, Ihre-Lundgren C, Hellman P. Primary small intestinal neuroendocrine tumors are highly prevalent and often multiple before metastatic disease develops. Scand J Surg 2019; doi: 10.1177/1457496919874484 [Epub ahead of print] 10.1177/1457496919874484 31587594

[16] Landerholm K , Falkmer S, Järhult J. Epidemiology of small bowel carcinoids in a defined population World J Surg 2010;34:1500–1505 20237925 10.1007/s00268-010-0519-z

[17] Ellis L , Shale MJ, Coleman MP. Carcinoid tumors of the gastrointestinal tract: trends in incidence in England since 1971. Am J Gastroenterol 2010;105:2563−2569 20823835 10.1038/ajg.2010.341

[18] Hauso Ø , Gustafsson BI, Kidd M, Waldum HL, Drozdov I, Chan AK et al Neuroendocrine tumor epidemiology: contrasting Norway and North America. Cancer 2008;113:2655−2664 18853416 10.1002/cncr.23883

[19] Lepage C , Bouvier A-M, Manfredi S, Dancourt V, Faivre J. Incidence and management of primary malignant small bowel cancers: a well-defined French population study. Am J Gastroenterol 2006;101:2826−2832 17026561 10.1111/j.1572-0241.2006.00854.x

[20] Lu Y , Fröbom R, Lagergren J. Incidence patterns of small bowel cancer in a population-based study in Sweden: increase in duodenal adenocarcinoma. Cancer Epidemiol 2012;36:e158–e163 22405637 10.1016/j.canep.2012.01.008

[21] Sandvik OM , Søreide K, Gudlaugsson E, Kvaløy JT, Søreide JA. Epidemiology and classification of gastroenteropancreatic neuroendocrine neoplasms using current coding criteria. Br J Surg 2016;103:226–232 26511392 10.1002/bjs.10034 PMC5061026

[22] Joo MK , Park JJ, Chun HJ. Proton pump inhibitor: The dual role in gastric cancer. World J Gastroenterol 2019;25:2058–2070 31114133 10.3748/wjg.v25.i17.2058 PMC6506576

[23] Cheung KS , Chan EW, Wong AYS, Chen L, Wong ICK, Leung WK. Long-term proton pump inhibitors and risk of gastric cancer development after treatment for *Helicobacter pylori*: a population-based study. Gut 2018;67:28–35 29089382 10.1136/gutjnl-2017-314605

[24] Bojesen RD , Andersson M, Riis LB, Nielsen OH, Jess T. Incidence of, phenotypes of and survival from small bowel cancer in Denmark, 1994–2010: a population-based study. J Gastroenterol 2016;51:891–899 26847562 10.1007/s00535-016-1171-7

[25] Legué LM , Bernards N, Gerritse SL, van Oudheusden TR, de Hingh IH, Creemers GM et al Trends in incidence, treatment and survival of small bowel adenocarcinomas between 1999 and 2013: a population-based study in the Netherlands. Acta Oncol 2016;55:1183–1189 27170100 10.1080/0284186X.2016.1182211

[26] Zar N , Holmberg L, Wilander E, Rastad J. Survival in small intestinal adenocarcinoma. Eur J Cancer 1996;32A:2114–2119 9014754 10.1016/s0959-8049(96)00244-4

[27] Zar N , Garmo H, Holmberg L, Rastad J, Hellman P. Long-term survival of patients with small intestinal carcinoid tumors. World J Surg 2004;28:1163−1168 15490058 10.1007/s00268-004-7610-2

[28] Landerholm K , Zar N, Anderson RE, Falkmer SE, Järhult J. Survival and prognostic factors in patients with small bowel carcinoid tumour Br J Surg 2011;98:1617–1624 21858790 10.1002/bjs.7649

[29] Buchbjerg T , Fristrup C, Mortensen MB. The incidence and prognosis of true duodenal carcinomas. Surg Oncol 2015;24:110–116 25936244 10.1016/j.suronc.2015.04.004

[30] Akce M , Jiang R, Zakka K, Wu C, Alese OB, Shaib WL et al Clinical outcomes of small bowel adenocarcinoma. Clin Colorectal Cancer 2019;18:257–268 31606297 10.1016/j.clcc.2019.08.002

[31] Randle RW , Ahmed S, Newman NA, Clark CJ. Clinical outcomes for neuroendocrine tumors of the duodenum and ampulla of Vater: a population-based study. J Gastrointest Surg 2014;18:354–362 24114680 10.1007/s11605-013-2365-4

